# Examining the Consequences of School Bans on the Self-esteem of Afghan Adolescent Girls in Kabul, Afghanistan

**DOI:** 10.1007/s40653-026-00919-x

**Published:** 2026-06-04

**Authors:** Fahima Sediqi

**Affiliations:** Graduate School of International Development, Nagoya University, Nagoya City, Japan

**Keywords:** Afghan adolescent girls, School bans, Self-esteem

## Abstract

Following the Taliban’s return to power in August 2021, they systematically banned Afghan girls from formal education beyond the sixth grade across the country, forcibly depriving millions of girls of educational opportunities in Afghanistan. This qualitative study explores the consequences of school bans on the psychological well-being and self-esteem of Afghan adolescent girls by examining how prolonged adversity following abrupt school bans, conceptualised as a potentially traumatic life experience, has affected Afghan adolescent girls’ daily routines, educational goals, and aspirations. The study employs semi-structured interviews and thematic analysis. The participants comprise 10 Afghan girls aged between 13 and 18 residing in Kabul. Data collection was conducted online from 17 August to 4 September, approximately three years after the imposition of school bans. Most participants had been experiencing school exclusion for three years. The findings indicate significant shifts in participants’ daily routines and psychological well-being, particularly among those from low socioeconomic backgrounds. Participants who experienced abrupt school exclusion reported lower psychological well-being, including increased social isolation, compared to those with prior awareness. In addition to the loss of agency experienced by the participants, the data analysis reveals stress-related somatic symptoms, including headaches, among a small number of participants. These findings emphasise the pressing need for psychological support and educational opportunities to mitigate the effects of prolonged adversity.

**Supplementary Information:**

The online version contains supplementary material available at https://doi.org/10.1007/s40653-026-00919-x.

Afghan girls’ educational condition deteriorated during the first period of the Taliban rule between 1996 and 2001. They were excluded from all forms of education, schools were destroyed, and the curriculum was restricted (BBC World Service, n.d.). However, after the overthrow of the Taliban due to the intervention of the United Nations and US forces in 2001, there was a significant increase in Afghan girls’ access to primary education. Overall, approximately 10 million girls were enrolled, up from about 1 million previously; the number of teachers increased by 58%, and the female literacy rate nearly doubled, rising from 17% to 30%. Growth in female enrolment in primary education was particularly noteworthy, increasing from almost none in 2001 to 2.5 million in 2018. Between 2001 and 2018, the number of girls enrolled in higher education rose from approximately 5,000 to 90,000 (UNESCO, 2021). Although Afghanistan’s educational system experienced rapid growth within various sections, challenges persisted. For instance, the quality of education, shortages of trained teachers, inadequate teacher salaries, and attacks on schools remained problematic, undermining progress and sustainability. Despite these obstacles, girls’ participation in education continued to grow, with significant efforts of the government and international organizations to promote equality and inclusive educational opportunities (Arooje & Burridge, 2020).

In 2021, unexpectedly, the Taliban regained power for the second time after the withdrawal of the United Nations and US military forces and the termination of their support in Afghanistan. Subsequently, the Taliban banned secondary education for girls in Afghanistan shortly (Fetrat, 2024). This situation changed Afghanistan into the only nation that prohibits girls and women from attending schools (UNESCO, 2024).

Notably, the formal bans on Afghan girls’ education beyond sixth grade, which marks the beginning of junior high school, coincides with the onset of adolescence, a crucial stage of human growth that includes rapid changes in self-esteem and social, physical, and cognitive growth (Biro et al., 2006). Girls’ access to education in Afghanistan has historically been a controversial issue, and Afghan girls have consistently faced discriminatory practices that directly undermine their worth and rights within family, society, culture, and traditional norms. Given the undeniable reality of Afghan girls’ experiences and the intensification of these challenges through gender-based school exclusion during the sensitive developmental period of adolescence, a few studies suggest that discrimination may be detrimental not only to adolescents’ everyday emotional and physical well-being but also to more stable psychological characteristics, including self-esteem (Huynh & Fuligni, 2010). Following the schools’ prohibitions, clinical observations suggest that the imposed school bans for adolescent girls coupled with limitations on mental health initiatives have made adolescent girls’ psychological issues worse. Significant levels of depression were observed among adolescent girls who were no longer attending school (Jobson et al., 2024). While there is little literature on the relationship between self-esteem and depression alongside key sociodemographic characteristics among school-going adolescents under Taliban control (Neyazi et al., 2023), the unique experiences of Afghan adolescent girls whose lives were initially disrupted by the school bans and subsequently shaped by the condition of prolonged adversity remain insufficiently studied.

This study aims to shed light on how the denial of Afghan adolescent girls’ identity as students and their access to schooling affects their self-esteem and psychological well-being. To address these gaps, the present study explores the following key research questions.

1. What is the consequence of school bans on the self-esteem of Afghan adolescent girls in Kabul, Afghanistan?

- How have Afghan adolescent girls’ daily lives been affected by school bans?
- How have Afghan adolescent girls’ long-term future careers and aspirations been altered since the school bans?
- How have these changes affected Afghan adolescent girls’ psychological well-being?

## Literature Review

### Self-esteem

Individuals’ motivation, emotional resilience, and mental health depend heavily on their self-esteem. According to Rosenberg (1965), self-esteem is an individual’s negative or positive attitude toward themselves and the extent to which that person believes that he is good enough and a person of worth. Greenwald et al. (1988) suggest self-esteem as the most significant component of self-concept. Two forms of self-esteem have remarkably distinct outcomes: specific self-esteem is more associated with behavior, whereas global self-esteem is more related to psychological well-being. Further research reveals that “A person’s self-esteem is fundamental to all behaviors and responses that are a part of the experiences they have created for themselves (Rosenberg et al., 1995). Low self-esteem can stem from negative self-concepts in areas deemed significant, especially when combined with rejection from close relationships (Harter, 2015). Individuals with low self-esteem struggle to balance their need for positive input with their tendency to doubt and distrust it. In contrast, individuals with high self-esteem have the desire to succeed, stand out, and positively impact others (Baumeister, 1997). Studies suggested that self-esteem is influenced by various factors, including culture, societal status, religion, and the messages we receive from people over time (Weber State University, n.d.).

### Self-esteem in Adolescence

Adolescence is universally recognized as a crucial stage of development, shaping individuals’ physical, emotional, and social well-being and laying the foundation for their future adult lives. “Adolescence is a developmental period characterized by a marked change in cognition, emotion, behavior, and contexts” (Moreira et al., 2015, p. 12). The period between childhood and adulthood, known as adolescence, lasts from ages 10 to 19 (WHO, n.d.). It is a distinct phase of human growth and a critical period for establishing a foundation for good health. This period is frequently characterized as a time of vulnerability and “increased risk for the onset of a wide range of emotional and behavioural problems including depression” (Steinberg, 2005, p. 69). There are five typical areas where kids and teenagers evaluate themselves: physical competence, scholastic competence, social competence, physical appearance, and behavioural conduct. (Harter, 1999). Adolescence is a crucial period for self-esteem development, with lasting effects on mental health and well-being (Steiger et al., 2014).

### Traumatic Life Experiences and Psychological Well-being

Several studies indicate that traumatic life experiences during adolescence, a developmental period marked by increased vulnerability to stress (Larson & Ham, 1993), may profoundly affect self-evaluative processes and identity development. Furthermore, such experiences often lead individuals to re-evaluate and question their commitments to goals, roles, beliefs, and values (Erikson, as cited in Berman, 2016). In this context, young people experiencing school exclusion may confront a range of interconnected traumas and family-related challenges that affect their educational engagement and future life trajectories (King, 2016). These adverse experiences are negatively associated with resilience and psychological well-being (Mahamid et al., 2020), which may have life-altering developmental implications for identity functioning during adolescence and emerging adulthood, ranging from resilience and self-affirmation to identity disruption and loss (Waterman, 2020).

### Schools and Psychological Well-being

Schools, beyond their function as learning centers, can contribute to promoting positive behavior and social interactions among students. According to Peterson (2006, as cited in Zeng et al., 2016), schools can function as positive institutions that focus on both academic achievement and student well-being. Furthermore, schools play a unique role in enhancing adolescents’ capacity to identify and establish the essential pathways for achieving their goals, thereby contributing to mental health promotion (Wilson & Somhlaba, 2016). Those with higher levels of education more frequently reported higher levels of eudaimonic and hedonic SWB. Similarly, it has also been pointed out that in most areas of life, those with more education report feeling more satisfied (Nikolaev, 2016). Conversely, some research suggests that the degree of education, whether primary, secondary, and tertiary, has no impact on the direct relationship between education and happiness (Cuñado & De Gracia, 2012).

## Method

### Conceptual Framework: Identity Theory

An identity is a collection of meanings that characterize individuals according to their roles, social groupings, or categories they are a part of, as well as the personal traits that set them apart from others. This can include role identity, such as being a student (a role identity), an honest individual (a personal identity), or a man or woman (a social identity). Verifying one’s role and personal identity improves one’s sense of worth and authenticity. Receiving confirmation of one’s identity, particularly when connected to moral principles or achievements, increases one’s sense of self-worth. This procedure, founded on fundamental ideas of identity theory, demonstrates how validating one’s self-concept and societal duties facilitates a stronger, more genuine sense of self (Stets & Burke, 2014).

Identity processes are disrupted by events that prevent individuals from aligning their self-perceptions with their identity standards, leading to distress and diminished feelings of mastery or efficacy, ultimately reducing self-esteem. According to identity theory, such disruptions in life are linked to adverse emotional outcomes, including distress and depression, as individuals struggle to regain control and maintain congruence between their identity and external feedback (Burke, 1991).

William James (1890) suggested that the ratio of success to aspirations determines an individual’s self-esteem.

$$\:Self-esteem=\frac{Successes}{Pretensions}$$

**Fig. 1:**
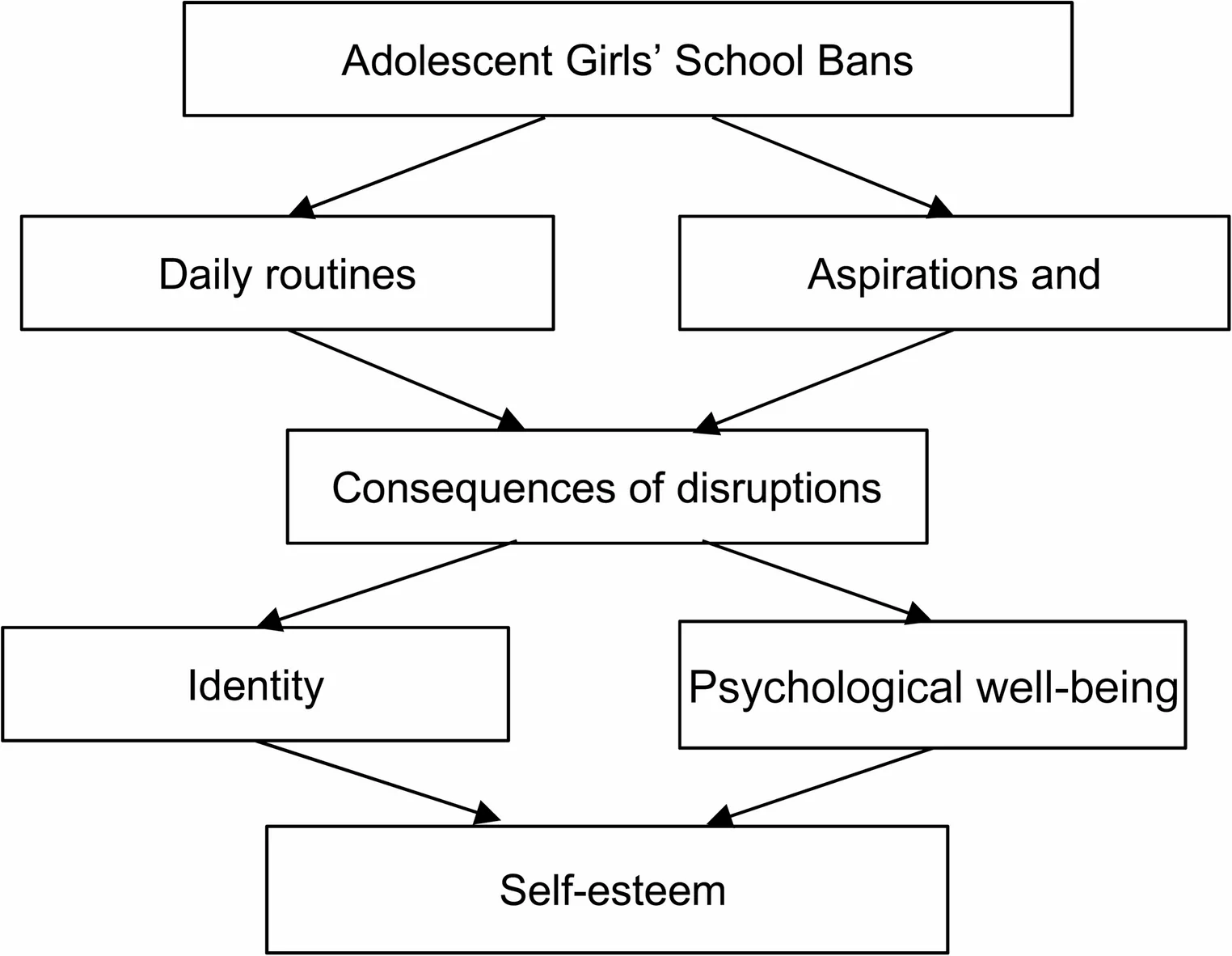
Conceptual framework of consequences of school bans. *Note*. Created by the author based on identity theory (Burke, 1991)

One’s self-esteem would be low if their dreams or pretensions outweighed their achievements. Small accomplishments may also result in strong self-esteem and the individual has lower expectations. Identity theories, which look at how well people match their true self-perceptions (“successes”) with their identity aspirations (“pretensions”), pointed out a close correspondence to William James’ notion of self-esteem. In identity verification, “successes” correspond to how individuals believe others perceive them in a particular circumstance, and “pretensions” correspond to individuals’ identity standards. Verifying identities within social groupings involves the role behaviors that uphold social structures and boost self-esteem. Self-verification enhances worth-based self-esteem by having others validate an individual’s role identification within a relationship or group. However, when individuals consistently fail to confirm their identification, their self-worth decreases, leaving them more vulnerable to adverse emotional impacts (Cast & Burke, 2002). The conceptual framework draws on identity theory (Burke, 1991; Cast & Burke, 2002; Stets & Burke, 2014) to explain how school bans and the undermining of Afghan adolescent girls’ student identity affect their psychological well-being and self-esteem through changes in their daily lives and the failure to fulfill their educational aspirations, as shown in Fig. 1.

### Research Approaches

The primary data source for this study’s analysis was informants’ interviews. To collect the primary data, semi-structured interviews were selected to provide flexibility in capturing distinctive insights while ensuring targeted investigation of important issues, such as social dynamics and self-esteem. This method makes a comprehensive understanding of Afghan adolescent girls’ experiences with school bans possible, maintaining consistency throughout interviews and being appropriate for the sensitive subject matter. Since this study aimed to explore how the school bans as a disruptive event have affected the daily routines, goals, and dreams of Afghan adolescent girls, thematic analysis is considered an appropriate approach that enables the researcher to perceive and interpret shared or collaborative meanings and experiences and identify relevant and meaningful responses to research questions (Braun & Clarke, 2012).

The participants were purposively selected from adolescent girls who were former pupils affected by the school bans. The sample consisted of ten key informants, aged 13–18, residing in Kabul province, identified with the assistance of the school’s teachers and head teacher, who acted as facilitators. In addition, the researcher’s prior experience as a former teacher contributed to access and established mutual trust with the participants and teaching staff. Parents were contacted before the interviews to seek their willingness and to explain the study’s purpose, ethical issues, and confidentiality. Prior to participation in the study, written consent forms were obtained from both parents and participants, including approval for the audio recording of interviews. This process ensured that both parents and participants were acquainted with the data collection procedures and their rights as research participants. Due to the sensitivity of the research topic, permission from the ethics committee was obtained; additionally, particular attention was paid to the potential emotional discomfort associated with the recollection of past educational exclusion and related life experiences.

The participants were informed in advance that some interview questions might evoke distress, and they were reminded of their right to refuse to answer any question or to withdraw from the study at any point without consequence. Whenever discomfort and emotional distress were detected, the interview was paused or redirected in order to minimise the emotional burden on the participants. No respondents elected to withdraw from or refuse to participate in the interviews.

Data collection was initiated from 17th August to 4th September via WhatsApp, the most widely used and accessible application in Afghanistan supporting text, voice, and video communication. Participants from higher and middle socioeconomic status (SES) groups preferred voice calls, whereas those from lower SES groups preferred video calls. Socioeconomic status (SES) was assessed using parental education, household income, and occupation. All interviews were scheduled to last approximately one hour; however, their duration varied depending on participants’ level of engagement and internet stability, which constituted one of the main challenges during the interview process. Consequently, participants were monitored at regular intervals (e.g., weekly) through phone calls and online meetings to assess their psychosocial well-being and ensure access to necessary support. No participants reported severe distress during or following the interviews. Table 1 presents the demographic characteristics of the research participants, highlighting the diversity of backgrounds and perspectives represented in the study.

**Table 1:** Demographic information of research participants

Respondents	Grade before the school bans	Age	Number of siblings	SES	Place of residence
Razia	9th	17	7	High	Kabul
Maryam	11th	18	6	High	Kabul
Mahtab	10th	17	6	Middle	Kabul
Sediqa	6th	15	6	Middle	Kabul
Diana	7th	15	9	Middle	Kabul
Taranom	10th	18	9	Low	Kabul
Nooria	7th	17	5	Low	Kabul
Basmeena	9th	17	9	Low	Kabul
Storai	6th	13	5	Low	Kabul
Arezo	7th	15	5	Low	Kabul

Data collected by the author through online interview conducted in September 2024

### Data Analysis

This study employed qualitative analysis to answer the research questions. The qualitative methodology adopted for this study was based on an interpretive paradigm, which stresses comprehending the individualized subjective experiences and meanings that individuals create. Utilizing a narrative inquiry, emphasizing the lived experiences and individual narratives of Afghan adolescent girls, the study sought to investigate the effects of school bans on their self-esteem. For data analysis, the researcher developed an Excel-based dashboard to systematically code transcribed interviews and interview notes, identifying and generating themes. The analysis followed the thematic analysis framework, a widely recognized method in qualitative research popularized by Braun and Clarke (2006), through six phases, combining deductive and inductive approaches.

Given the sensitive nature of the participants’ experiences, the audio recordings were not shared with additional reviewers due to sensitive nature of research and to maintain strict confidentiality and uphold ethical safeguards. Instead, analytical rigor was strengthened through iterative listening to recordings and continuous reflexive engagement with the data to capture their essence, ensuring the nuances and meanings were accurately understood. The interview transcripts were human-translated from Dari to English without utilizing software to generate a more direct translation, which would better preserve the source text’s nuance. After transcription, the six phases of thematic analysis outlined by Braun and Clarke (2006) were employed to analyse the data systematically.

## Results

The analysis of Afghan adolescents’ experiences led to the identification of five central themes that structured the participants’ narratives after experiencing school bans: (1) disruption of daily routines; (2) disruption of aspirations and educational trajectories; (3) loss of agency and emotional distress; (4) social isolation and emotional withdrawal; and (5) resilience and coping strategies demonstrated by adolescent participants. Through thematic analysis, guided by identity and self-esteem theories, subthemes emerged that revealed the profound subjective impact of school bans on Afghan adolescent girls’ lives, future orientations, and psychological well-being.

### Disruption of Daily Routines

According to the participants’ statements, before the school bans, their mornings were filled with lessons and the promise of a bright future through regularly engaging in various formal and informal educational activities within and beyond their homes. The schools and educational centers had provided numerous participants with a unique ground for personal growth, making it a routine and predictable aspect of their day.

Before the school ban, I would attend Madrassa every morning, studying until 8:00 AM and teaching others until 10:00 AM. Then, I would go to school and return home around 2:00 PM. Afterward, I would attend additional courses before heading home. (Taranom, 18)

However, the adolescent participants’ daily lives drastically altered to an unstructured pattern following the imposition of school bans.

When they closed the schools, we had to stay at home. The English and math courses that we used to go were shut down too, now we are stuck at home and there is nothing to study and nothing to do. (Basmeena, 17)

The adolescents’ narratives highlight that before the school closures, they regularly interacted with peers as part of their daily routines and maintained a consistent presence within their communities.

Before the school bans, we were much more involved in society and communicated frequently with our friends and those around us. (Razia, 17)

Nevertheless, participants’ peer relationships were adversely affected after school closure, particularly those that were established through interactions at school and within their neighbourhood settings in order to practice the daily learning activities.

I don’t have any updates about my friends. We used to regularly get together at school and in courses to review and practice our lessons and assignments; however, everyone is currently busy with their own lives, so neither they nor we come to visit each other. (Taranom, 18)

The findings reveal that school bans contributed to a significant decline in peer interaction and social engagement, which were essential elements of the adolescent participants’ daily routines. This limitations led to the loss of their identities as students and learners, which were previously validated and reinforced by their access to schools.

#### Socioeconomic Status as a Determinant of Daily Educational Routines

The analysis of participants’ experiences indicates that school bans intensified the role of socioeconomic status in shaping their daily lives. Although schools remained closed, many girls attempted to maintain their educational routines through informal learning initiatives, including offline and online platforms, as alternatives to sustain their identities as students. However, access to these resources was largely dependent on their families’ financial capacity.

Since we can’t afford the same schools and courses as the other girls, my sister and I usually go to the stationery store to purchase books and study on our own. Every day, I study English through YouTube platforms and study Islamic books, such as the Holy Quran, at home. (Sediqa, 15)

Approximately half of the respondents from low socioeconomic backgrounds reported significant alterations in their daily routines. Inability to access alternative learning resources due to financial limitations resulted in their complete exclusion from educational activities.

When the schools were banned, we got stuck at home and could no longer participate because the fees were too high. No longer I can do those activities that I did before. (Diana, 15)

Consequently, these disadvantaged participants shifted their focus toward supporting their families through traditional gender role activities such as housework and handicrafts.

I do some embroidery on scarves, for which we get paid 40–50 Afghanis each. I also do pearl embroidery. (Storai, 13)

Notably, several participants’ parents lost their jobs after the Taliban took back control, which made their financial situation worse. These economic constraints further limited the girls’ ability to maintain consistent daily routines, particularly in relation to their educational engagement.

Under the previous regime, my father could work, and our economic condition was better, allowing us to attend courses. Our current financial situation prevents us from accessing alternative learning opportunities, such as paid courses and Madrassa education. (Basmeena, 17)

Deprivation from school not only prevented girls from pursuing their education but also exacerbated disparities in access to alternative learning resources. Access to such opportunities was highly dependent on financial capacity, which had previously been less significant due to the availability of free public education.

### Disruption of Aspirations and Educational Goals

The striking findings from the respondents highlight that before the imposition of school bans, adolescent participants aspired for a future shaped by their academic ambitions and successes. Almost all respondents in the sample expressed their prior strong desire and intention to complete their schooling and enroll in universities.

After the educational restrictions, I shifted my path and dream from medicine to technology, telling myself “Let’s pursue another path if the previous one is no longer possible.” (Maryam, 18).

Before the school ban, I wished to complete my schooling and receive my school certification, then attend university, study there, and become a doctor. (Basmeena, 17)

Although financial constraints limited educational opportunities for participants from middle-income households, they actively sought alternative learning resources, including free or low-cost options to pursue their educational goals. As a result, some participants engaged in online educational programs offered by non-governmental organizations. Nevertheless, various challenges gradually undermined their confidence in these alternative learning pathways and in achieving their aspirations through these platforms. As one participant reported:

I decided to attend online classes and pursue my studies, but after a while, I realized that they were of low quality, and I could not learn. Most of the students who had fallen behind took part. In addition, the Ministry of Education does not recognize the current courses. Even if we study at home, we don’t gain anything from it because we’re not hopeful, since for the past three years, they have been saying that schools will reopen. (Mahtab, 17)

Similarly, another participant from the same background was following an English language course. She was determined to gain skills to work in language institutions in the future and contribute to helping other girls overcome environmental and financial constraints to learning.

I am studying to achieve something significant in the future to support others by launching free courses to teach them so that they can learn. If I can to pursue my studies and learn, I can help and give back this knowledge to others. (Arezo, 15)

On the other hand, the aspirations of adolescent girls from lower socioeconomic settings, were severely constrained by limited resources and inadequate support. As a result, looking forward to school reopening was the only solution and practical way for these disadvantaged participants to continue their educational trajectories and fulfill their aspirations.

The findings suggest that prolonged adversity severely disrupted the girls’ previously planned career trajectories, creating a break in the continuity of their educational and personal development. This interruption generated a sense of mourning for the selves they could have become under normal circumstances. The majority of participants, especially those who experienced sudden school exclusion and prolonged adversity lasting more than three years, expressed consistent accounts of regret and loss due to their inability to achieve their educational aspirations, resulting in a pervasive sense of unfulfillment.

I could have graduated by now if I had been able to continue… [Pause]… we had dreams and goals…but we could not fulfil them [Voice breaking]. (Razia, 17)

Despite adjusting to the reality and adopting a different path, one of the participants never fully gave up on her initial desire. Her broken dreams and unfulfilled ambitions that once shaped her future career as a part of her identity are still alive in her heart and mind.

Even now, whenever I see a doctor, visit one, or watch a movie where a doctor appears, hearing the word ‘doctor’ revives the feelings I had buried, and it awakens the despair of knowing I could have been a doctor, or I wish I could be a doctor. (Maryam, 18)

### Loss of Agency under Prolonged Adversity

The analysis of narratives reveals a complex and ongoing process of loss of agency experienced by adolescent girls under the prolonged adversity of educational exclusion. The school bans were configured as a cumulative trauma that disrupted identity formation, future orientation, and perceived personal control. These narratives echoed well-established trauma processes, including learned helplessness, which were further intensified by the erosion of social validation and support, resulting in persistent emotional distress and dissatisfaction.

Bans on schools have made my life chaotic and disorganized, and I do not know what to do. (Taranom, 18)

When I was able to attend school, I could do daily workouts. I knew about the process of my life and growing up, and my current level. However, now, I am stuck at home all day, and I am stressed out. (Maryam, 18)

The majority of participants identified the first day of the academic year, following several months of enforced absence from school, as a profoundly distressing moment.

My most painful memory is the day when the Taliban came and, after one year, recalled us to school. However, they recalled us only to formally announce that we were no longer allowed to attend school. They told us that we were completely prohibited from continuing our education. This remains the most painful memory for me, and whenever I recall it, it makes me deeply sad. (Razia, 17)

Participants’ narratives suggest that these experiences remain deeply embedded in memory, with recollection triggering the same distressing and potentially traumatic responses.

One of the participants expressed that the prolonged school bans and her inability to make independent decisions about her personal life and educational trajectory led her to lose a sense of purpose.

Bans on schools have made my life chaotic and disorganized, and I do not know what to do. (Taranom, 18)

The denial of participants’ identity as students in society created a profound contradiction between the girls’ self-perception and their envisioned selves shaped through educational aspirations, resulting in a loss of control over current adversity and experiencing feelings of dissatisfaction.

Life is unfair. What is a dream for us now is something ordinary for others. My life is not satisfying, because I’m unable to achieve my aspiration. It is an impossible aspiration. My friends and I are not as happy as we used to be in the past. (Mahtab, 17)

The adolescent participants had already determined their long-term and short-term objectives; they already dreamed of their future careers. However, the forced and abrupt school bans disrupted their purpose in life and left them with a sense of dissatisfaction.

We are currently neither happy nor satisfied. In the past, when we could attend school, we were delighted, but now we are not. I cannot do anything anymore. I have no way. (Basmeena, 17)

According to respondents’ narratives, early marriage has become a major concern for many participants. Schools and access to education functioned as a crucial protective shield against girls’ early and forced marriage; however, the absence of these opportunities and the increase in gender inequality have intensified girls’ vulnerability to early marriage. As a result, many participants struggle with the fear of facing a similar fate.

Many were forced into marriage because there was no opportunity for education, so what else could they do? They were forced into marriage as children without any education, and this makes me anxious. I am always overwhelmed and nervous when I am thinking of the same fate for myself, leaving me distressed. (Nooria, 17)

Repeated exposure to prolonged school bans and uncontrollable restrictions appeared to foster trauma-related responses consistent with learned helplessness, whereby participants came to perceive their efforts as ineffective in shaping outcomes. Several adolescent participants expressed their desperation about their futures.

Our future… (sigh and pause). I gradually became frustrated and hopeless, leading me to believe that we might never return to our schools and studies. The Taliban keep promising that schools will reopen, but … [pause] we do not believe their words anymore. Our future … [pause, sigh]. (Razia, 17)

For my future, I feel entirely desperate if the Taliban remains in power. We have no future with them in control, and when they leave, maybe… [Sigh] We still do not have any hope… [Pause]. (Basmeena, 17)

Several adolescents reported experiencing stress-related physical symptoms in response to uncertainty surrounding school reopening and repeated disappointing news.

I watch TV every day, but the news is often filled with disappointing and concerning remarks about girls being unable to attend school. They say that girls must not go to school, and it makes me feel so exhausted that I cannot sleep at night. I’m struggling with headaches. This condition has worsened, and it’s especially bad at night when I want to sleep. (Taranom, 18)

Prolonged exposure to school bans and stressful daily conditions resulted in distressing experiences, including both physical and mental health issues among several adolescent participants. These symptoms were further exacerbated as participants engaged with news and community narratives, heightening their awareness of uncertainty about current circumstances and the possibility of future changes.

### Social Isolation and Emotional Withdrawal

The findings indicate that prior to school exclusion adolescent participants were consistently present in the community and were able to interact regularly with peers. Schools were safe sanctuaries where adolescent participants could freely communicate, share their goals and dreams with peers, and receive regular self-verification through their student roles.

When we were in school, my classmates and I would study together and support each other. We could always meet up and share our learning experiences. (Arezo, 15)

However, prolonged and forced disconnection led to profound feelings of loneliness and isolation. Participants’ narratives further revealed that schools functioned as safe environments where girls anchored social bonds and a sense of belonging.

After school bans, I feel more isolated because we cannot go anywhere, making it impossible to make new friends. Being stuck at home has left me with a deep sense of loneliness and isolation. (Nooria, 17)

One of the respondents reported that although she made multiple attempts to contact her friends, she discovered a significant shift in both her and her friends’ interpersonal communication.

I noticed some changes in myself and them. Intimacy decreased, maybe because of the distance; now, people have distanced themselves from each other and become isolated and unsociable. I noticed my friends are busier with social media like Instagram and TikTok and do not interact with others. (Maryam, 18)

At the same time, a few adolescent respondents reported familial restriction when attempting to go out or interact with their friends.

Before, we could meet and talk to our friends at school or even go to the park. But now, since there are no schools, we are not allowed to go out. Whenever we try, our parents question us, asking where we’re going and who we’re with. (Sediqa, 15)

While many participants expressed feelings of social isolation and loneliness, this participant expressed that she was able to overcome emotional withdrawal and regain a sense of belonging through her re-engagement with society and participation in collaborative activities with her peers.

After attending the Atarud^1^ course for seven months in person, I rebuilt my self-confidence and became more sociable. Before that, I did not talk much and did not feel happy. In addition, by participating in the development online program for the last six months, we, as four members, had a chain that made me say that when they have not given up yet, I will not either. I moved forward with them and completed the projects for the sake of each other. It means that I realized that I am not alone. (Maryam, 18)

Notably, her socioeconomic background facilitated access to these opportunities, enabling her to afford the resources to participate fully in such activities.

### Erosion of Social Validation and Support

Many participants reported that their parents continuously provided emotional support and inspired them to pursue their academic goals before the school prohibitions.

My dad always encouraged me to read poems and reassured me that I could achieve what I liked. He kept telling me I would get a first grade at university because of my hard work and endeavours. (Mahtab, 17)

The participants’ narratives revealed that many of these respondents, particularly those in the later years of secondary schooling, had already shaped their goals and aspirations in alignment with their families’ expectations and preferred career paths.

Before schools were prohibited, my mother always told me that I would become a doctor or a teacher, and she was incredibly proud of the idea of me becoming a teacher. (Taranom, 18)

However, following the imposition of school bans, several respondents expressed a significant decline in the regular positive feedback and encouragement they once received from their parents. This shift disrupted self-verification, undermining their confidence and motivation to pursue their goals and aspirations.

[…] but after the school bans, he never told me again as before and never said, “You can, and you will succeed in doing what you want” any longer. It made me think that until now, my father accompanied me; however, from this time onwards, there is no one with me any longer. (Mahtab, 17)

The inability to fulfill parental expectations profoundly affected these young participants’ psychological well-being, leading to feelings of inadequacy and self-blame.

When my parents see a girl who has achieved something on social media, I can see the despair on their faces, especially in my dad’s eyes. They both had high hopes and expectations for our future. My dad worked hard and sacrificed so much for me. He never said anything to make us feel we could not meet those expectations. (Maryam, 18)

While one of the participants acknowledged the emotional weight of her mother’s words, she refrained from blaming her; instead, she pointed to the systemic barriers imposed by the Taliban’s educational restrictions.

My mom believes that educational qualifications and a 12th-grade completion certificate are essential everywhere. She said, “It has been three years, and you still don’t have a certificate; you can’t study or become a teacher now.” Her words make me anxious and distressed, but I cannot blame her, because this situation was caused by the Taliban. My parents have always wanted us to succeed in education. (Taranom, 18)

The findings highlight that adolescent participants became aware of the gap between their aspirational standards and existing realities through prolonged exposure to school bans; as a result, this externally imposed adversity led them to experience negative feelings of unfulfillment and failure. Moreover, the lack of positive parental feedback functioned as a disruption in their identity verification process, leaving them with doubts about their sense of worth and efficacy. Although participants’ parents no longer explicitly pointed to the loss of aspirations, the emotional weight of both verbal and nonverbal expressions remained deeply imprinted in their minds.

In this context, the public’s negative attitude toward adolescent respondents’ dreams and goals emerges as a significant contributor to feelings of inferiority and self-doubt about their worth and rights. This societal mindset minimizes their aspirations and reinforces barriers to personal and academic growth, creating a cycle of diminished confidence and restricted opportunities.

Our neighbouring women and relatives tell us, “Getting married is your destiny, so follow it.” Sometimes, their words are very offensive, saying, “You could study until sixth grade.” People, including our relatives, use offensive and mocking words like, “Instead of twelfth-grade certificates, you have sixth-grade ones. (Taranom, 18)

### Social Comparison

Participants’ narratives revealed that school bans intensified social comparison among adolescent participants. During the interviews, all respondents consistently compared themselves with girls both inside or outside Afghanistan and boys who could attend school freely.

Boys’ schools are open, and my brothers can attend, although they are uninterested. In contrast, we were eager to participate in school, but the school’s doors have been shut. (Basmeena, 17)

I compared myself with my friends and relatives abroad; when I was talking and asking about their activities, and they said they were studying and doing this or that, it made me think, ‘They can study while I have nothing to do.’ (Maryam, 18).

The persistent gender-based discrimination and inequitable access to education contributed to a diminishing sense of self-efficacy among participants. Furthermore, evolving societal attitudes toward adolescent girls undermined their sense of self-worth.

### Adaptive responses: Coping and resilience

Although participants experienced a profound disruption in their daily life, goals, and future aspirations, they demonstrated remarkable resilience through employing various coping strategies to mitigate the toll of emotional distress. These coping strategies are significantly influenced by their socioeconomic status and family support. Those from higher and middle socioeconomic backgrounds pursued various available educational opportunities and focused on enhancing their skills to prepare for future careers. Indeed, these efforts provided them a sense of continuity and purpose.

Besides studying, reading books, and watching Harry Potter movies, I have set specific goals to keep myself busy. I created conditions for myself; for instance, if I study for two days in preparation for an interview and complete the material, I reward myself by allowing time to watch a movie. I have also decided to give myself small gifts to stay motivated. I exercise for 20–30 min daily and set weekly and monthly goals, which I review regularly to assess my achievements through regular checking and taking notes. (Maryam, 18)

Another respondent from a middle socioeconomic background continues to review her schoolbooks, using them to envision and stay connected to her school experience.

I keep reviewing and studying my textbooks, hoping my school will reopen one day. Doing this makes it feel like I am still going to school and learning, even if it is just in my mind. (Sediqa, 15)

In contrast, participants from low socioeconomic backgrounds engaged in handicrafts and household responsibilities to remain occupied, temporarily redirect their attention from their reality, and support their families. Although they were unable to afford educational resources, these activities slightly contributed to reducing their emotional distress.

I make myself busy by engaging in housework to forget or change my thoughts and process my grief. (Diana, 15)

I do pearl embroidery, and these activities have helped shift my mood, keeping my mind off things and bringing a small amount of happiness by staying busy. (Basmeena, 17)

A few respondents reported daily workouts that provided them with a sense of relief.

I am accustomed to taking an early-morning walk. It makes me feel comfortable and helps to redirect my attention. (Basmeena, 17)

Furthermore, faith and spirituality served as a key source of strength and inspiration, enabling participants to remain hopeful about the future and the eventual reopening of schools.

Despite not being allowed to participate in school, I hope the schools will reopen one day, and we can study there. I trust in Allah, and maybe one day, schools will reopen. (Diana, 15)

One of the participants continues to dream of the day when she will be allowed to attend school freely, obtain her certificate, and pursue her aspirations. Occasionally, her brothers engage in playful teasing or share inaccurate information about school reopening, which sometimes results in moments of happiness for her.

Sometimes, my brothers come home from school and, as a joke, tell us, ‘Tomorrow, you will be able to go to school because they reopened.’ Even though it is not true in those moments, it fills us with joy. I dream of the day when schools reopen, and we can study again, proudly achieve our certificates, say goodbye to our teachers, and freely pursue our goals at university. (Taranom, 18)

## Discussion

The forced and abrupt disruptions to the daily lives of Afghan adolescent participants resulted in a diminished sense of control and decision-making. Furthermore, this prolonged adversity exacerbated feelings of distress and helplessness among these marginalized girls. According to identity theory, individuals strive to align their self-perceptions with their identity standards. When external circumstances prevent this alignment, such as being denied access to education, individuals experience disruptions in their identity processes, leading to distress, lower mastery, and low self-esteem (Burke, 1991). The findings highlight that adolescent participants experienced profound negative psychological consequences following school exclusion and drastic disruptions to their daily lives. Trauma literature suggests that such experiences may have life-altering developmental implications for identity functioning during adolescence, a developmental period marked by intensified vulnerability to stress (Larson & Ham, 1993; Waterman, 2020).

In addition, the socioeconomic status of adolescent girls significantly contributed to shaping their daily activities and adopting new goals and paths for their future after implementing school bans. Compared to respondents from middle and low SES backgrounds in the sample, participants from higher SES backgrounds could pursue various educational opportunities as part of their daily routines and adopt new goals and paths toward their future through consistent support from their families, which positively affected respondents’ self-esteem. This finding contradicts the findings of Neyazi et al. (2023), which pointed out that self-esteem was considerably lower among teenagers from high-income households, followed by middle-class adolescents and then low SES adolescents, possibly due to factors such as the unavailability of emotional support from parents.

The findings reveal that participants from low socioeconomic backgrounds were left behind in terms of educational opportunities and became primarily engaged in daily housework and handicraft activities. This finding supports previous reports that life disruptions would particularly affect adolescents in low-income households (Von Soest et al., 2022). Considering that male students often report higher self-esteem levels than female students (Khan et al., 2019), the findings highlight how discriminatory practices can undermine the self-esteem of girls.

William James’s (1890) concept of the ratio of success to pretension offers a valuable perspective. According to this framework, individuals’ self-esteem is shaped by the extent to which their achievements (successes) align with their aspirations (pretensions). In this context, the girls’ aspirations to pursue education and enter university were significantly constrained, creating a gap between their identity aspirations and their lived reality. The findings suggest that this disruption and the inability to pursue their educational aspirations might negatively undermine the adolescent participants’ self-perception.

Studies indicate that identity verification, the process of receiving confirmation from others about one’s identity, plays a significant role in shaping self-esteem. The narratives of participants highlight their perseverance in accessing alternative learning opportunities to validate their identities within their families and communities. Despite their remarkable efforts, they struggled with a lack of external recognition and confirmation. Given that self-esteem is shaped through the identity verification process (Cast & Burke, 2002), this lack of validation could undermine the development of positive self-esteem. Furthermore, identity theory pointed out that disruptions in identity verification, such as being denied access to education, could have significant emotional consequences. The girls’ experiences reveal the profound experience of losing social feedback and the opportunity to validate their roles, emphasizing the critical role of societal recognition in shaping their identities and self-worth.

The negative psychological consequences of teenagers’ life disruption have been exacerbated due to the lack of community and family social support, coupled with changes in their behavior following the imposition of educational bans that connect ideas of “having people like family members or close friends to turn in times of need or crisis to provide care and help promote a positive self-image” (Mao & Agyapong, 2021, p. 7). Traumatic life experiences following the school bans, have left adolescent girls with negative emotions such as feelings of anxiety, stress, and depression, which aligns with the findings of Magson et al. (2021).

The study reveals a severe sense of unfulfillment and loss among participants, particularly those who experienced abrupt school bans and were in the later years of secondary education. They had already established structured goals and career plans for the future as an adult. They envisioned achieving their aspirations shortly, which is consistent with the findings of Viejo et al. (2018) and supports the findings of Neyazi et al. (2023) that 94% of adolescents from high school reported low self-esteem due to their future educational concerns and university entrance.

The adolescent girls sought happiness and life satisfaction in the school degree and educational certificate, which contrasts with Cuñado and De Gracia’s (2012) findings. The study’s respondents displayed varying levels of resilience influenced by their socioeconomic status and personal characteristics to cope with trauma and mitigate negative emotions; however, they struggle with feelings of hopelessness regarding their future and education under the current regime. This contrasts with Karatas and Cakar’s (2011) findings, which suggest that decreased hopelessness enhances resilience. Moreover, inequality in access to alternative learning resources significantly affected adolescent girls from disadvantaged backgrounds in the sample. It may make them more prone to low self-esteem (Veselska et al., 2010), which is also profoundly affected by sociocultural and socioeconomic factors. In Afghanistan’s patriarchal society, traditional gender roles and escalating restrictions on girls’ rights and freedoms erode their sense of self-worth. These gendered barriers, coupled with limited educational opportunities, foster feelings of failure and unfulfillment, shattering their dreams and goals and perpetuating a sense of inferiority that hinders their personal growth.

## Conclusion

This study explored the consequences of school bans imposed after the Taliban’s return to power in 2021 on the self-esteem and psychological well-being of Afghan adolescent girls and examined how educational exclusion disrupted their daily lives, aspirations, and future-oriented goals. The findings indicate that the exclusion from schools profoundly affected adolescents’ routines and their attainment of future goals, particularly for those from a disadvantaged background. In addition, the findings underscore the long-term trauma-related developmental consequences of prolonged adversity, particularly for Afghan adolescent girls who experienced abrupt and unanticipated school bans. Long-term exposure to gender-based exclusion and discriminatory practices may intensify the vulnerability of adolescent girls, placing them at greater risk of long-term psychosocial consequences.

### Limitations of Study

This study has several limitations. First, interviews were conducted online with the participants in Afghanistan, where unstable internet connectivity led to occasional interruptions of interview sessions. Second, participants’ reluctance to engage in video calls limited the researcher’s ability to observe nonverbal cues, such as facial expressions and physical reactions, which may have enriched emotional interpretation and analysis. The use of online interviews may also have constrained participants’ comfort and emotional openness, particularly when discussing sensitive or traumatic experiences. Additionally, political and cultural sensitivities, combined with limited access to private spaces, may have contributed to the underreporting of emotional distress due to participants’ hesitancy or safety concerns. Finally, while this study provides valuable insights into the experiences of Afghan adolescent girls under school bans, further research is needed to examine the long-term psychological consequences of this prolonged educational exclusion during adolescence.

### Recommendations

This study highlights the urgent need for support and intervention to address the severe trauma and disruptions caused by school bans on Afghan adolescent girls. To inform such interventions, future research should adopt a mixed-methods approach, combining qualitative interviews and quantitative surveys, with a preference for in-person or hybrid interviews to overcome technical barriers and enhance comfort. Further, the findings reveal significant disparities in access to learning resources based on socioeconomic status and a decline in the well-being of disadvantaged girls. Providing affordable self-study materials and fostering skills such as digital literacy, critical thinking, and emotional resilience would significantly contribute to empowering these girls to pursue education and prepare for future opportunities. A multifaceted approach addressing both immediate and long-term barriers is essential for personal and societal progress.

## Supplementary Information

Below is the link to the electronic supplementary material.

Supplementary Material 1

Supplementary Material 2

Supplementary Material 3

## Data Availability

The interview data supporting the findings of this study are not publicly available due to ethical and privacy restrictions but are available from the corresponding author upon reasonable request.

## Footnotes

1 Atarude is a well-known practical English institute in Kabul, Afghanistan.

## References

[CR2] Arooje, R., & Burridge, N. (2020). School education in Afghanistan: Overcoming the challenges of a fragile state. In P. M. Sarangapani & R. Pappu (Eds.), *Handbook of Education Systems in South Asia* (pp. 1–33). Springer Singapore. 10.1007/978-981-13-3309-5_10-1

[CR3] Baumeister, R. F. (1997). Identity, self-concept, and self-esteem. In R. Hogan, J. Johnson, & S. Briggs *Handbook of Personality Psychology* (pp. 681–710). Academic Press. 10.1016/B978-012134645-4/50027-5

[CR4] BBC World Service. (n.d.). *Article 26: Right to education: Case study: Education in Afghanistan*. https://www.bbc.co.uk/worldservice/people/features/ihavearightto/four_b/casestudy_art26.shtml

[CR5] Berman, S. L. (2016). Identity and trauma. *Journal of Traumatic Stress Disorders and Treatment*, *5*(2), 1–4. 10.4172/2324-8947.1000e108

[CR6] Biro, F. M., Striegel-Moore, R. H., Franko, D. L., Padgett, J., & Bean, J. A. (2006). Self-esteem in adolescent females. *Journal of Adolescent Health*, *39*(4), 501–507. 10.1016/j.jadohealth.2006.03.010 16982384

[CR7] Braun, V., & Clarke, V. (2006). Using thematic analysis in psychology. *Qualitative Research in Psychology*, *3*(2), 77–101. 10.1191/1478088706qp063oa

[CR8] Braun, V., & Clarke, V. (2012). Thematic analysis. In H. Cooper, P. M. Camic, D. L. Long, A. T. Panter, D. Rindskopf, & K. J. Sher (Eds.), *APA Handbook of Research Methods in Psychology, Vol. 2: Research Designs: Quantitative, Qualitative, Neuropsychological, and Biological* (pp. 57–71). American Psychological Association.

[CR9] Burke, P. J. (1991). Identity processes and social stress. *American Sociological Review*, *56*(6), 836–849. 10.2307/2096259

[CR10] Cast, A. D., & Burke, P. J. (2002). A theory of self-esteem. *Social Forces*, *80*(3), 1041–1068. 10.1353/sof.2002.0003

[CR11] Cuñado, J., & de Gracia, F. P. (2012). Does education affect happiness? Evidence for Spain. *Social Indicators Research*, *108*(1), 185–196. 10.1007/s11205-011-9874-x

[CR12] Fetrat, S. (2024). *Taliban’s attack on girls’ education harming Afghanistan’s future*. Human Rights Watch. https://www.hrw.org/news/2024/09/17/talibans-attack-girls-education-harming-afghanistans-future

[CR14] Greenwald, A. G., Bellezza, F. S., & Banaji, M. R. (1988). Is self-esteem a central ingredient of the self-concept? *Personality and Social Psychology Bulletin*, *14*(1), 34–45. 10.1177/0146167288141004 30045451

[CR15] Harter, S. (1999). *The Construction of Self: A Developmental Perspective*. Guilford Press.

[CR16] Harter, S. (2015). Self-development in childhood and adolescence. In J. D. Wright (Ed.). *International Encyclopedia of the Social & Behavioral Sciences* (pp. 492–497). Elsevier. 10.1016/B978-0-08-097086-8.23019-5

[CR17] Huynh, V. W., & Fuligni, A. J. (2010). Discrimination hurts: The academic, psychological, and physical well-being of adolescents. *Journal of Research on Adolescence*, *20*(4), 916–941. 10.1111/j.1532-7795.2010.00670.x

[CR18] James, W. (1890). *The principles of psychology*. Henry Holt and Company.

[CR19] Jobson, L., McAvoy, D., & Ahmadi, S. J. (2024). Psychological problems in the context of political violence in Afghan children. *Current Psychiatry Reports*, *26*(5), 222–228. 10.1007/s11920-024-01496-2 PMC11081985 38564145

[CR20] Karatas, Z., & Cakar, F. S. (2011). Self-esteem and hopelessness, and resiliency: An exploratory study of adolescents in Turkey. *International Education Studies*, *4*(4), p84. 10.5539/ies.v4n4p84

[CR21] Khan, I., Mahmood, A., & Zaib, U. (2019). Interplay of self-esteem with the academic achievements between male and female secondary school students. *Journal of Human Behavior in the Social Environment*, *29*(8), 971–978. 10.1080/10911359.2019.1611517

[CR22] King, H. (2016). The connection between personal traumas and educational exclusion in young people’s lives. *YOUNG*, *24*(4), 342–358. 10.1177/1103308815627752

[CR23] Larson, R., & Ham, M. (1993). Stress and storm and stress in early adolescence: The relationship of negative events with dysphoric affect. *Developmental Psychology*, *29*(1), 130–140. 10.1037/0012-1649.29.1.130

[CR24] Magson, N. R., Freeman, J. Y. A., Rapee, R. M., Richardson, C. E., Oar, E. L., & Fardouly, J. (2021). Risk and protective factors for prospective changes in adolescent mental health during the COVID-19 pandemic. *Journal of Youth and Adolescence*, *50*, 44–57. 10.1007/s10964-020-01332-9 PMC7590912 33108542

[CR26] Mahamid, F., Bdier, D., & Chou, P. (2020). Traumatic life events and psychological well-being among Palestinian adolescents: The mediating role of resilience. *Journal of Concurrent Disorders*, *3*(3), 105. 10.1007/s44202-023-00087-1-117

[CR25] Mao, W., & Agyapong, V. I. O. (2021). The Role of social determinants in mental health and resilience after disasters: Implications for public health policy and practice. *Frontiers in Public Health*, *9*, 658528. 10.3389/fpubh.2021.658528 PMC8170026 34095062

[CR45] Moreira, P. A. S., Cloninger, C. R., Dinis, L., Sá, L., Oliveira, J. T., Dias, A., & Oliveira, J. (2015). Personality and well-being in adolescents. *Frontiers in Psychology, 5,* Article 1494. 10.3389/fpsyg.2014.01494 PMC4285735 25610408

[CR27] National Institute of Mental Health. (2024). *Coping with traumatic events*. https://www.nimh.nih.gov/health/topics/coping-with-traumatic-events

[CR28] Neyazi, A., Padhi, B. K., Ahmadi, M., Rangelova, V., Mohammadi, A. Q., Neyazi, M., & Griffiths, M. D. (2023). Depression and self-esteem among Afghan school-going adolescents under the Taliban Government: A cross-sectional study. *Discover Psychology*, *3*, 25. 10.1007/s44202-023-00087-1

[CR29] Nikolaev, B. (2016). Does higher education increase hedonic and eudaimonic happiness? *Journal of Happiness Studies*, *19*, 483–504. 10.1007/s10902-016-9833-y

[CR31] Rosenberg, M. (1965). *Society and the adolescent self-image*. Princeton University Press.

[CR30] Rosenberg, M., Schooler, C., Schoenbach, C., & Rosenberg, F. (1995). Global self-esteem and specific self-esteem: Different concepts, different outcomes. *American Sociological Review*, *60*(1), 141–156. 10.2307/2096350

[CR33] Steiger, A. E., Allemand, M., Robins, R. W., & Fend, H. A. (2014). Low and decreasing self-esteem during adolescence predict adult depression two decades later. *Journal of Personality and Social Psychology*, *106*(2), 325–338. 10.1037/a0035133 24467425

[CR34] Steinberg, L. (2005). Cognitive and affective development in adolescence. *Trends in Cognitive Sciences*, *9*(2), 69–74. 10.1016/j.tics.2004.12.005 15668099

[CR35] Stets, J. E., & Burke, P. J. (2014). Self-esteem and identities. *Sociological Perspectives*, *57*(4), 409–433. 10.1177/0731121414536141

[CR37] UNESCO. (2021). *UNESCO sounds a warning on what is at stake for education in Afghanistan*. https://www.unesco.org/en/articles/unesco-sounds-warning-what-stake-education-afghanistan

[CR38] UNESCO. (2024). *Afghanistan: 1.4 million girls still banned from school by de facto authorities*. https://www.unesco.org/en/articles/afghanistan-14-million-girls-still-banned-school-de-facto-authorities

[CR39] Veselska, Z., Madarasova Geckova, A., Gajdosova, B., Orosova, O., Van Dijk, J. P., & Reijneveld, S. A. (2010). Socio-economic differences in self-esteem of adolescents influenced by personality, mental health and social support. *European Journal of Public Health*, *20*(6), 647–652. 10.1093/eurpub/ckp210 20034930

[CR40] Viejo, C., Gómez-López, M., & Ortega-Ruiz, R. (2018). Adolescents’ psychological well-being: A multidimensional measure. *International Journal of Environmental Research and Public Health*, *15*(10), 2325. 10.3390/ijerph15102325 PMC6210683 30360456

[CR41] von Soest, T., Kozák, M., Rodríguez-Cano, R., Fluit, S., Cortés-García, L., Ulset, V. S., Haghish, E. F., & Bakken, A. (2022). Adolescents’ psychosocial well-being one year after the outbreak of the COVID-19 pandemic in Norway. *Nature Human Behaviour*, *6*(2), 217–228. 10.1038/s41562-021-01255-w 35058644

[CR42] Waterman, A. S. (2020). Now what do I do? Toward a conceptual understanding of the effects of traumatic events on identity functioning. *Journal of Adolescence*, *79*(1), 59–69. 10.1016/j.adolescence.2019.11.005 31901704

[CR43] Weber State University. (n.d.). *Self-Esteem*. Consulting and Psychological Services Center. Retrieved April 29, 2026, from https://www.weber.edu/counselingcenter/self-esteem.html

[CR1] Wilson, A., & Somhlaba, N. Z. (2016). Psychological well-being in a context of adversity: Ghanaian adolescents’ experiences of hope and life satisfaction. *Africa Today*, *63*(1), 85. 10.2979/africatoday.63.1.0085

[CR46] World Health Organization. (n.d.). *Adolescent health.* https://www.who.int/health-topics/adolescent-health

[CR44] Zeng, G., Hou, H., & Peng, K. (2016). Effect of growth mindset on school engagement and psychological well-being of Chinese primary and middle school students: The mediating role of resilience. *Frontiers in Psychology, 7*, Article 1873. 10.3389/fpsyg.2016.01873 PMC5147462 28018251

