# Supplementary material for: Examining the Consequences of School Bans on the Self-esteem of Afghan Adolescent Girls in Kabul, Afghanistan
Source: J Child Adolesc Trauma. 2026 Jun 4;19(4):2381–94. doi: 10.1007/s40653-026-00919-x (PMC13737272; doi:10.1007/s40653-026-00919-x)
Supplement: Supplementary file 1 [file 40653_2026_919_MOESM1_ESM.pdf]

Parent/Guardian Consent Form

Objectives: We are currently conducting a study entitled "Investigating the Experiences of Afghan Adolescent Girls Banned from School and the Impact on Their Self-Esteem in Kabul, Afghanistan." This study aims to examine the experiences of Afghan adolescent girls affected by official school bans and to explore how these restrictions have influenced their daily lives, educational goals, and aspirations. Furthermore, the study investigates the effects of these disruptions on their psychological well-being and self-esteem.

Procedure: Your daughter is invited to participate in this study as one of the girls affected by school closures. If you agree, she will participate in a semi-structured interview focusing on her experiences with school closures and their impact on her daily life, social interactions, educational goals, and future aspirations. The interview will last approximately one hour and will be audio-recorded with your permission to ensure accurate capture of responses.

Risks and Discomforts: There are no significant physical risks associated with this study. However, some interview questions may cause emotional discomfort, as participants may recall difficult experiences related to school closures. She may choose not to answer any questions that make her feel uncomfortable or may skip any questions at any time without explanation. Participation in this study is voluntary, and your daughter may withdraw from the interview at any time without any negative consequences

Confidentiality: All information collected in this study will be kept confidential. Your daughter's data will be anonymized, and no identifying information will be disclosed in any reports or publications. Access to the research data will be restricted to the researcher and, where necessary, the research supervisor or ethics committee for review purposes.

Ethics: This study was conducted in accordance with relevant institutional regulations and national ethical guidelines for research involving human subjects in Japan and was reviewed and approved by an institutional ethics committee.

Consent: By signing below, you confirm that you have read and understood the information provided above, that you voluntarily give permission for your daughter to participate in this interview, and you consent to the interview being audio-recorded.

Name of Participant's Parent/Guardian: ___________________
Signature of Parent/Guardian: ___________________
Date: ___________________
