# Supplementary material for: Examining the Consequences of School Bans on the Self-esteem of Afghan Adolescent Girls in Kabul, Afghanistan
Source: J Child Adolesc Trauma. 2026 Jun 4;19(4):2381–94. doi: 10.1007/s40653-026-00919-x (PMC13737272; doi:10.1007/s40653-026-00919-x)
Supplement: Supplementary file 2 [file 40653_2026_919_MOESM2_ESM.pdf]

Participants' Consent Form

Objectives: We are currently conducting a study entitled "Investigating the Experiences of Afghan Adolescent Girls Banned from School and the Impact on Their Self-Esteem in Kabul, Afghanistan." This study aims to examine the experiences of Afghan adolescent girls affected by official school bans and to explore how these restrictions have influenced their daily lives, educational goals, and aspirations. Furthermore, the study investigates the effects of these disruptions on their psychological well-being and self-esteem.

Procedure: As one of the girls affected by the school bans, you are invited to participate in this study to answer a set of semi-structured questions. These questions will focus on your experiences with the school bans and their impact on your daily life interactions and future educational goals. The interview will last approximately one hour and will be audio recorded with your consent to ensure your comments are accurately captured.

Risks and Discomforts: There are no significant physical risks associated with this study. However, some questions may cause emotional discomfort because they involve recalling experiences related to school bans. You may choose not to answer any questions that make you feel uncomfortable, and you may skip any questions at any time without giving a reason. Your participation is voluntary, and you may withdraw from the study at any time without any negative consequences.

Confidentiality: All information collected in this study will be kept confidential. Your data will be anonymized, and no personal or identifying information will be shared. Access to the data will be restricted to the researcher and, where necessary, to the research supervisor or the ethics committee for review purposes. You are encouraged to answer openly and honestly to the extent that you feel comfortable.

Ethics: This study was conducted in accordance with relevant institutional regulations and national ethical guidelines for research involving human subjects in Japan and was reviewed and approved by an institutional ethics committee.

Consent: By signing below, you indicate that you have read and understood the information provided above. You voluntarily agree to participate in the interview and consent to the audio recording of the interview.

Name of Participant: ____________________

Signature of Participant: ____________________

Date: ____________________
