# Supplementary material for: Examining the Consequences of School Bans on the Self-esteem of Afghan Adolescent Girls in Kabul, Afghanistan
Source: J Child Adolesc Trauma. 2026 Jun 4;19(4):2381–94. doi: 10.1007/s40653-026-00919-x (PMC13737272; doi:10.1007/s40653-026-00919-x)
Supplement: Supplementary file 3 [file 40653_2026_919_MOESM3_ESM.pdf]

Research Questions

1. How and where were you first informed about the school bans?
2. In which grade were you no longer allowed to attend school?
3. How did you feel when you realized that you could no longer attend school?
4. Can you describe your daily routine before and after the school bans?
5. Do you feel that your life has changed since the school closures? If so, how?
6. What has been your family's response to the school closure for girls?
7. Have roles and expectations within your family changed since the school bans?
8. Have your family members or relatives supported you since the school bans? If so, what kind of support have they provided?
9. Since the school bans, have you noticed any changes in how people view girls' education? If so, what are your thoughts and feelings about these changes?
10. Has the school ban affected your friendships and social interactions?
11. Have you found new ways to connect with friends?
12. Are you currently involved in any alternative forms of education or learning?
13. How do you feel about these alternative learning methods compared to formal schooling? Do you think they are effective?
14. What were your dreams and aspirations for the future before the school bans?
15. Did the school bans change your goals or aspirations for the future? How?
16. How do you feel when you are unable to pursue the future you hoped for?
17. How have the challenges in pursuing your goals affected the way you feel about yourself?
18. Do you feel differently about your abilities since the school bans? If so, how?
19. What activities or strategies have helped support your happiness and emotional well-being?
20. Have you found any new sources of inspiration or support since the school bans?
21. How do you maintain hope and motivation for your future?
22. How do you cope with the feelings associated with not being allowed to attend school?
